# Dental Surgery in the Royal College of Surgeons

**Published:** 1872-11

**Authors:** 


*Dental Surgery in the Royal College of Surgeons.*—The "*London Lancet,*" informs us that an examination for the licence in Dental Surgery of the Royal College of Surgeons of England has been held during the last week, and is remarkable for being the first occasion on which a written test has been introduced into the examination. On Friday, the 21st inst., the candidates had placed before them two papers, one on general Anatomy and Surgery, and one on Dental Anatomy and Surgery; and on Tuesday, the 25th, a *viva voce* examination was held by the members of the Dental Board, consisting of Messrs. Partridge, Hilton, Busk, Cartwright, Ibbetson, and Salter. We are happy to hear that all the candidates were successful on the present occasion, and that the written papers were of considerable merit. The dental diploma carries with it, by a recent regulation of the Council, the right of admission to the library and museum and to the lectures of the College of Surgeons.

